# Baseline Neuroimaging Predicts Decline to Dementia From Amnestic Mild Cognitive Impairment

**DOI:** 10.3389/fnagi.2021.758298

**Published:** 2021-12-07

**Authors:** Joseph M. Gullett, Alejandro Albizu, Ruogu Fang, David A. Loewenstein, Ranjan Duara, Monica Rosselli, Melissa J. Armstrong, Tatjana Rundek, Hanna K. Hausman, Steven T. Dekosky, Adam J. Woods, Ronald A. Cohen

**Affiliations:** ^1^Department of Clinical and Health Psychology, University of Florida, Gainesville, FL, United States; ^2^Department of Neuroscience, University of Florida, Gainesville, FL, United States; ^3^Clayton J. Pruitt Department of Biomedical Engineering, University of Florida, Gainesville, FL, United States; ^4^Center for Cognitive Neuroscience and Aging, University of Miami Miller School of Medicine, Miami, FL, United States; ^5^Department of Neurology, University of Florida, Gainesville, FL, United States; ^6^Department of Psychology, Florida Atlantic University, Davie, FL, United States; ^7^Department of Neurology, University of Florida, Gainesville, FL, United States; ^8^Evelyn F. McKnight Brain Institute, Department of Neurology, University of Miami Miller School of Medicine, Miami, FL, United States

**Keywords:** machine learning, support vector machine, magnetic resonance imaging, mild cognitive impairment, Alzheimer’s disease

## Abstract

**Background and Objectives:** Prediction of decline to dementia using objective biomarkers in high-risk patients with amnestic mild cognitive impairment (aMCI) has immense utility. Our objective was to use multimodal MRI to (1) determine whether accurate and precise prediction of dementia conversion could be achieved using baseline data alone, and (2) generate a map of the brain regions implicated in longitudinal decline to dementia.

**Methods:** Participants meeting criteria for aMCI at baseline (*N* = 55) were classified at follow-up as remaining stable/improved in their diagnosis (*N* = 41) or declined to dementia (*N* = 14). Baseline T1 structural MRI and resting-state fMRI (rsfMRI) were combined and a semi-supervised support vector machine (SVM) which separated stable participants from those who decline at follow-up with maximal margin. Cross-validated model performance metrics and MRI feature weights were calculated to include the strength of each brain voxel in its ability to distinguish the two groups.

**Results:** Total model accuracy for predicting diagnostic change at follow-up was 92.7% using baseline T1 imaging alone, 83.5% using rsfMRI alone, and 94.5% when combining T1 and rsfMRI modalities. Feature weights that survived the *p* < 0.01 threshold for separation of the two groups revealed the strongest margin in the combined structural and functional regions underlying the medial temporal lobes in the limbic system.

**Discussion:** An MRI-driven SVM model demonstrates accurate and precise prediction of later dementia conversion in aMCI patients. The multi-modal regions driving this prediction were the strongest in the medial temporal regions of the limbic system, consistent with literature on the progression of Alzheimer’s disease.

## Introduction

While the clinical course of Alzheimer’s disease (AD) is fairly well-understood, the ability to predict progression from an earlier stage of the disease using data available upon initial clinical presentation remains poor. With the advancement of machine learning, clinicians are now presented with the opportunity to identify which high-risk patients are likely to convert to AD, such as those diagnosed with amnestic mild cognitive impairment (aMCI) (Petersen, 2004). This ability to provide early identification of at-risk patients additionally has a large medical-economic cost savings given that early intervention to delay the onset of Alzheimer’s by just 1 year, for example, could reduce total health care payments up to 14% (Zissimopoulos et al., 2015) and decrease the number of Alzheimer’s diagnoses by 9.2 million by 2050 (Brookmeyer et al., 2007).

Patients are given a diagnosis of aMCI when they demonstrate a delayed memory performance score that is 1.5 standard deviations or more from the mean of their like-aged peers (Petersen et al., 2014). The conversion rate to dementia in patients with aMCI ranges from as low as 17.7% in community-derived samples, up to 40.4% in clinic samples (Oltra-Cucarella et al., 2018), regardless of follow-up length. This is compared to 5.4–10.1% of “all” MCI cases and < 1% in healthy older adults in community-derived samples over a 5 year period (Ganguli et al., 2015, 2019). Machine learning models utilizing Support Vector Machines (SVM) offer enhanced predictive accuracy for disease progression by integrating previously uncharacterized features of multiple neuroimaging modalities with or without the addition of cognitive performance data to distinguish between two groups of patients (Ruppert, 2004), such as those who convert from MCI to AD and those who remain classified as MCI. In recent years, prediction of disease progression from MCI to dementia or presumed AD has been explored with SVM using baseline MRI measures of all structural voxels (Moradi et al., 2015), cortical thickness (Eskildsen et al., 2013), cortical and subcortical volume (Hojjati et al., 2018), and resting-state fMRI (rsfMRI) connectivity (Li Y. et al., 2016) in isolation, with prediction accuracies of 66, 76, 89, and 93%, respectively.

Given that the amount of data at an initial clinical visit is often quite limited, a model that could provide strong predictive accuracy of MCI conversion to AD using MRI-alone would be of immense utility. To our knowledge, only one other study has used a combined model of structural and resting-state functional MRI to predict all MCI conversion to AD (Hojjati et al., 2018). Replication of this study in an aMCI population would provide unique information about this higher-risk population, as well as offer the ability to derive the neural regions where structural and functional networks combined to predict conversion from aMCI to AD. Ultimately, acquisition of the combined structural-functional neural regions important for conversion to AD would provide the opportunity for early neurotherapeutic interventions in high-risk aMCI patients.

In the present study, we sought to leverage baseline T1 MRI in a homogenous sample of individuals diagnosed with aMCI to predict longitudinal consensus-based diagnostic decline using a cross-validated SVM approach. Further, we wished to determine whether the inclusion of an additional MRI modality (resting-state functional MRI; rsfMRI) into the prediction model would improve predictive accuracy of the uni-modal structural model. Lastly, we sought to determine if neuropsychological performance at baseline outperformed objective neuroimaging for the prediction of longitudinal diagnostic decline. We hypothesized that T1 MRI would have a higher level of predictive accuracy than rsfMRI when used individually, but that the combination of these two modalities would provide the highest level of predictive accuracy. Further, we hypothesized that the combined structural-functional model would yield neural regions in the medial temporal lobes underlying the limbic network that would optimally discriminate stable aMCI from progressive aMCI, given the lower network connectivity in MCI compared to controls (Li et al., 2015) as well as the strong association of this network with the presence of Alzheimer’s disease (Badhwar et al., 2017). Lastly, given the use of many of the neuropsychological test measures in the determination of the aMCI or dementia diagnosis being predicted, we hypothesized that neuropsychological testing would outperform neuroimaging in the prediction of future decline.

## Materials and Methods

### Participant Selection

Participants were recruited through the 1Florida Alzheimer’s Disease Research Center (ADRC) for an IRB-approved longitudinal investigation performed in accordance with the declaration of Helsinki (P50-AG047266-05). Participants from the present study were selected from a larger pool of 287 potential participants if they met the following criteria: (a) valid T1 and rsfMRI neuroimaging scans at baseline, (b) consensus diagnosis of either single-domain or multi-domain amnestic MCI at baseline alone, (c) had no other neurological or cognitive diagnoses (e.g., Parkinson’s disease, suspected Lewy Body Dementia, vascular dementia) at baseline, (d) consensus diagnosis available at both baseline and follow-up, (e) no aberrant QC metrics of rsfMRI data at baseline to include greater than +/−3 SD values for in-scanner movement, global correlation of connectivity due to motion, or number of invalid scans.

### Participant Diagnosis

An experienced geriatric psychiatrist administered a standard clinical assessment protocol, which included the CDR^®^ Dementia Staging Instrument (CDR) (Morris, 1997) and the Montreal Cognitive Assessment (MoCA) (Nasreddine et al., 2005). Subsequently, a uniform battery of neuropsychological tests, including the National Alzheimer’s Coordinating Center -Unified Data Set (NACC-UDS) (Beekly et al., 2007; Acevedo et al., 2009; Weintraub et al., 2018) battery, was independently administered in the participant’s dominant and preferred language (English or Spanish). Participants received a diagnosis of amnestic mild cognitive impairment (aMCI) at the baseline assessment if they met Petersen’s criteria for MCI (Petersen et al., 2014) and demonstrated all of the following: (a) subjective cognitive complaints by the participant and/or collateral informant; (b) evidence by clinical evaluation or history of memory or other cognitive decline; (c) Global Clinical Dementia Rating scale of 0.5 (Morris, 1997); (d) below expected performance on delayed recall of the HVLT-R (Brandt, 1991) or delayed paragraph recall from the Logical Memory subtest of the NACC-UDS (Beekly et al., 2007) as measured by a score that is 1.5 SD or more below the mean using age, education, and language-related norms. Participants were classified as multi-domain amnestic MCI if they met the above criteria as well as ≤1.5 SD performance on at least one other domain measure. All of these standard criteria were reviewed by an experienced behavioral neurologist (RD) or a board-certified neuropsychologist (DL). All these criteria were reviewed by a neurologist and a neuropsychologist and using an algorithmic diagnosis procedure final clinical diagnoses were made (Duara et al., 2010, 2011). In the few cases where consensus could not be obtained, at least one additional neurologist and neuropsychologist were consulted to render a final cognitive diagnosis.

For the purposes of determining diagnostic change at the follow-up visit, participants must have participated in the above assessment at least one calendar year (mean = 15.45 months; range 12.0–17.0 months) subsequent to their initial visit, which must have included acquisition of their whole-brain MRI. To be determined as “stable” in their diagnosis, the follow-up visit consensus diagnosis must be either the same (aMCI) or mildly improved (pre-MCI). For the purposes of this classification, pre-MCI diagnosis (see Loewenstein et al.,2012) included the following: (a) subjective memory complaints by the participant and/or or collateral informant; (b) evidence by clinical evaluation or history of memory or other cognitive decline determined after an extensive CDR interview); (c) Global CDR scale of 0.5; (d) a neuropsychological battery (see below) was deemed normal by a clinical neuropsychologist and generally, no measures in the neuropsychological battery fell 1.0 SD or more below normal limits, relative to age and education related normative data. To be considered “declined” in their diagnosis, the follow-up visit consensus diagnosis must have been determined as Dementia per the criteria a and b as described for the aMCI group above, and evidenced all of the following: (a) Global CDR score of 1.0; (b) below expected performance on the memory measures described above that scored 2.0 SD or more below the mean using age, education, and language-related norms.

### Neuropsychological Battery

Participants completed a comprehensive neuropsychological evaluation which assessed various cognitive domains. Verbal memory was measured using the HVLT-R (Brandt, 1991; Arango-Lasprilla et al., 2015b) and Craft 21 Story Recall (Craft et al., 1996); confrontation naming was assessed with the MINT (Gollan et al., 2012); visuospatial cognitive functioning was evaluated with the Benson Figure Drawing (Possin et al., 2011) and Block Design (Wechsler et al., 2008); executive function was appraised with the Stroop Test (Stroop, 1935; Trenerry et al., 2012; Tobergte and Curtis, 2013), as well as TMT B (Reitan, 1958; Arango-Lasprilla et al., 2015a); and finally, verbal fluency was assessed using category (Benton, 1968; Ostrosky-Solis et al., 2007) and phonemic fluency (Ruff et al., 1996).

Spanish language evaluations were completed with equivalent standardized neuropsychological tests. Tasks administered to primary Spanish speakers had appropriate age, education, and cultural/language normative data for the translated versions (Lang et al., 2021). Testing was performed by proficient Spanish/English psychometricians.

### Magnetic Resonance Imaging

Participants completed a 1-h MRI acquisition on a Siemens Skyra 3 T MRI scanner (Siemens Medical Solutions, Erlangen, Germany) with 32-channel head coil at Mount Sinai Medical Center, Miami Beach, Florida. The 3D T1 weighted volumetric magnetization-prepared rapid gradient-echo sequence (MP-RAGE) consisted of 176 slices at slice thickness = 1 mm isotropic, FOV = 256 × 256, TR = 3.0 s, and TE = 1.4 s. The resting-state functional MRI (rsfMRI) scan was administered with eyes open consisting of 48 interleaved slices at a slice thickness = 3.0 mm isotropic, FOV = 212 × 212, TR = 3.0 s, and TE = 30 ms. For exclusionary purposes of potential incidental findings, MRI scans were evaluated by visual inspection as well as with T2 weighted FLAIR (5 mm thick sequential axial slices), and the MP-RAGE sequence (which provides high tissue contrast and high spatial resolution with whole brain coverage).

#### Functional Magnetic Resonance Imaging Pre-processing

Functional MRI pre-processing was completed in accordance with past studies by our group (Hausman et al., 2020). Specifically, functional images were preprocessed and analyzed using the MATLAB R2019b based functional connectivity toolbox “Conn toolbox” version 18b and SPM 12 (Penny et al., 2007; Whitfield-Gabrieli and Nieto-Castanon, 2012). We followed a pre-processing pipeline which included functional realignment and unwarping, functional centering of the image to (0, 0, 0) coordinates, slice-timing correction, structural centering to (0, 0, 0) coordinates, structural segmentation and normalization to MNI space, functional normalization to MNI space, and spatial smoothing with a kernel of 8 mm FWHM. During pre-processing, the Conn toolbox implements an anatomical, component-based, noise correction strategy (aCompCor) for spatial and temporal processing to remove physiological noise factors from the data (Behzadi et al., 2007). The implementation of aCompCor combined with the quantification of participant motion and the identification of outlier scans through the Artifact Rejection Toolbox (ART) allows for better interpretation of functional connectivity results (Behzadi et al., 2007; Whitfield-Gabrieli and Nieto-Castanon, 2012; Shirer et al., 2015). The ART was set to the 97th percentile setting with the mean global-signal deviation threshold set at *z* = ± 3 and the participant-motion threshold set at 0.9 mm. Applying linear regression and using a band-pass filter of 0.008–0.09 Hz, data were de-noised to exclude signal frequencies outside of the range of expected BOLD signals (such as low-frequency scanner drift), minimize participant motion, extract white matter and cerebral spinal fluid noise components, and control for within-participant realignment and scrubbing covariates.

#### Structural Magnetic Resonance Imaging Pre-processing

Individual T1-weighted images were converted from DICOM to NIFTI using dcm2niix (Li X. et al., 2016). T1 images were then skull-stripped and transformed with the into MNI space using the default Conn processing pipeline for anatomical volumes, which utilizes MNI-space direct normalization (Whitfield-Gabrieli and Nieto-Castanon, 2012). Manual inspection of skull-stripping performance was completed to ensure optimal brain extraction for each subject. To reduce potential bias introduced by automated segmentation procedures, all voxels of the skull-stripped, MNI-normalized, T1-weighted data for each subject were included into the model, with regional analyses being performed subsequent to feature extraction (described below).

### Supervised Machine-Learning

Within- and between-network connectivity calculations were performed using ROI-ROI analyses of the 7-network Thomas Yeo et al. (2011) parcellation atlas. Functional connectivity of each connection was input as the pairwise connectivity of the 51 parcellations of the seven Thomas Yeo et al. (2011) atlas networks, which is calculated via Fisher z-transformed bivariate correlations between brain regions’ BOLD time-series that quantify associations in the activation at rest. Redundant pairs were removed to result in a final total of 1,275 connections. Participant classes were determined by separating participants into binary groups based on maintenance or decline in consensus diagnostic criteria at the follow-up visit most proximal to the diagnosis of aMCI. Due to the high dimensionality of MRI data, feature selection was performed on the training data to further reduce the number of trained features. One popular method of feature selection is to filter the features via voxelwise *t*-tests between classes to select current elements with a significant group-level difference (*p* < 0.01) as features for the subsequent prediction step (Iguyon and Elisseeff, 2003; Saeys et al., 2007; Dubois et al., 2018). Due to the difference in unit scale between the T1 and rsfMRI images, the selected features were standardized via z-score transformation. To classify stable participants and those who declined, we used SVM; a machine learning algorithm to search for the optimal hyperplane that separates two classes with maximal margin under the assumption of independently and identically distributed (iid) data (Andreola, 2009), which is satisfied in this study. Specifically, LIBSVM (Chang and Lin, 2011) was used to optimize the objective function:


$$\min_{w,b}\frac{1}{2}\,w^{T}\,w+c\,\sum_{i=1}^{i}\text{max}\,{\left(1-y_{i}\,\left(w^{T}\,x_{i}+b\right),0\right)}^{2}$$

where C is a penalty parameter on the training error. In other words, to address the issue of unbalanced data, the penalty parameter, C, was proportionally scaled for the minority class (i.e., greater penalty for incorrect classification of decline class compared to stable). A linear kernel was generated with the function:


$$K\,\left(x_{i},x_{j}\right)=x_{i}^{T}\,x_{j}$$

Model performance was evaluated across 10 permutations of two-level nested stratified cross-validation (Lindquist et al., 2017; Varoquaux et al., 2017; Polosecki et al., 2020). To elaborate, we began by splitting the data into randomized folds and performed an outer cross-validation loop consisting of k iterations. In each iteration, leave-one-out cross-validation was used to separate a single test case per fold in an outer loop. An inner stratified cross-validation loop was then performed on the training data (*N* = 54) with 10-folds, providing an optimal hyper-parameter C. A voxel-level *t*-test on T1w signal intensity/functional connectivity values within each cross-validation fold (i.e., 55 times) was performed on the training data only. Following training, predictions of held out test data were performed with the decision function:


$$f\,\left(x\right)=s\,g\,n\,\left(w^{T}\,x+b\right)$$

As a sub-investigation of the effect of single-domain aMCI and multi-domain aMCI on prediction outcomes, model performance was further evaluated as above after separating subgroups with single-domain impairment (*N* = 23) and multi-domain impairment (*N* = 32). In other words, the above model was evaluated for its ability to predict diagnostic decline at follow-up in patients with single-domain impairment, and again in patients with multi-domain impairment.

Lastly, to assess the predictive capabilities of baseline neuropsychological data (see Table 1), we employed identical SVM procedures as above to predict aMCI decline to dementia. Both a class-mean filling approach and a list-wise deletion approach were compared in their ability to handle neuropsychological data missing at random (MAR). As a note, when removing cases with missing data, further decreased group balance was observed and as such, we proportionally adjusted the penalty parameter C (as above) to account for the unbalanced data prior to running the final SVM model.

**TABLE 1:** Demographics and cognitive performance at baseline for total sample, consensus diagnosis change, and single- vs. multi-domain amnestic MCI groups.

	**Total (*N* = 55)**	**Stable at follow-up^f^ (*N* = 41)**	**Decline at follow-up^f^ (*N* = 14)**	***p*-value**
Age	72.5 (7.7)	72.0 (6.6)	73.8 (10.3)	0.466
Education	15.0 (3.14)	14.9 (3.0)	15.3 (3.5)	0.735
Gender (% Female)	56.4	53.7	64.3	0.489
Race (% White)	94.5	95.1	92.9	0.612
Hispanic (%)	54.5	53.7	57.1	0.821
Spanish first language (%)	40.0	41.5	35.7	0.743
Follow-up length (months)	15.45 (3.56)	16.92 (4.89)	14.95 (2.89)	0.173
CDR SOB^a^	1.17 (0.59)	0.98 (0.51)	1.71 (0.47)	<0.001
CDR global^b^	0.50 (0.0)	0.50 (0.0)	0.50 (0.0)	–
Hippocampal atrophy (%)^d^	54.5	51.2	64.3	0.765
APOE positive (%)^e^	25.5	24.4	28.6	0.140
Single-domain aMCI (%)	41.8	51.2	14.3	0.016
Multi-domain aMCI (%)	58.2	48.8	85.7	0.016
**Cognitive performance**				
MoCA total score	22.0 (3.0)	22.6 (2.9)	20.1 (3.0)	0.084
HVLT-R delayed recall	1.8 (3.3)	1.6 (2.9)	3.00 (4.1)	0.493
Craft story delayed recall	13.2 (7.0)	15.2 (6.5)	7.8 (1.8)	0.005
MINT naming	25.9 (5.3)	26.1 (4.1)	23.5 (7.6)	0.260
Benson figure drawing	15.3 (1.3)	15.5 (1.1)	14.5 (1.8)	0.163
Trail-making test, Part B	138.8 (68.5)	125.4 (63.9)	178.3 (68.5)	0.011
Semantic fluency	15.4 (4.4)	16.2 (4.2)	13.0 (4.2)	0.017

	**Total (*N* = 55)**	**Single-domain aMCI (*N* = 23)**	**Multi-domain aMCI (*N* = 32)**	***p*-value**

Age	72.5 (7.7)	72.2 (7.8)	72.8 (7.8)	0.782
Education	15.0 (3.14)	14.9 (3.0)	15.1 (3.3)	0.808
Gender (% Female)	56.4	56.5	56.3	0.984
Race (% White)	94.5	95.7	93.8	0.242
Hispanic (%)	54.5	65.2	46.9	0.178
Spanish first language (%)	40.0	47.8	43.8	0.262
Follow-up length (months)	15.45 (3.56)	15.3 (3.0)	15.6 (3.9)	0.736
CDR SOB^a^	1.17 (0.59)	0.91 (0.6)	1.36 (0.6)	0.005
CDR global^b^	0.50 (0.0)	0.50 (0.0)	0.50 (0.0)	−
Hippocampal atrophy (%)^d^	54.5	39.1	65.6	0.103
APOE positive (%)^e^	25.5	13.0	34.4	0.107
**Cognitive performance**				
MoCA total score	22.0 (3.0)	23.6 (3.3)	21.1 (2.5)	0.021
HVLT-R delayed recall	1.8 (3.3)	2.14 (4.0)	1.64 (2.8)	0.605
Craft story delayed recall	13.2 (7.0)	17.5 (5.7)	10.7 (6.6)	0.005
MINT naming	25.9 (5.3)	28.3 (3.5)	24.6 (5.8)	0.031
Benson figure drawing	15.3 (1.3)	15.8 (1.3)	15.0 (1.3)	0.125
Trail-making test, Part B	138.8 (68.5)	110.8 (50.2)	161.9 (75.1)	0.005
Semantic fluency	15.4 (4.4)	16.9 (3.8)	14.3 (4.5)	0.032

*^a^Clinical Dementia Rating Scale Sum of Boxes at baseline.*

*^b^Clinical Dementia Rating Scale global score at baseline.*

*^c^Positron Emission Tomography (PET) imaging.*

*^d^Neurologist confirmed on T1 MRI.*

*^e^Apolipoprotein E-4 allele present.*

*^f^Based on the NACC UDS Consensus Diagnosis.*

### Statistical Analysis

After all k iterations in the outer cross-validation loop were performed, predicted labels of all participants were compared against ground truth labels to calculate performance metrics. A Precision-Recall curve of positive predictive value against true positive rate was plotted to demonstrate the separability of classes within each model by calculating the area under the curve (AUC). The F1 score was generated given that it (1) takes both precision and recall into account to ultimately measure the accuracy of the model while accounting for false positives and false negatives, (2) is often more useful in models with unequal groups, such as the present study. Essentially, the F1 score ranges from 0 to 1 and gives more weight to false negatives and false positives while not letting large numbers of true negatives influence the score, which is helpful in dichotomous prediction models such as the present study. A high F1 score (e.g., over 90%) means that the model has limited false positives and false negatives, indicating the model has correctly identified real threats while not being disturbed by false alarms. Lastly, the Matthew’s correlation coefficient (MCC) was also calculated for each modality given that it may represent a more reliable statistical approach in binary prediction models that achieve good results in all possible outcomes (Chicco and Jurman, 2020).

### Functional Regions of Interest

The 7-network Thomas Yeo et al. (2011) parcellation atlas was utilized for determination of regions of interest (ROIs). In this atlas, the seven main networks include the Cingulo-Opercular Network (consisting of the parietal operculum, temporal occipital cortex, frontal operculum, lateral prefrontal cortex), Default Mode Network (prefrontal cortex, posterior cingulate cortex, parahippocampal cortex, and parietal and temporal cortices (corresponding to the angular gyrus and middle temporal gyrus, posterior division, respectively), the Dorsal Attention Network [posterior cortex (corresponding to the lateral occipital cortex, superior division), frontal eye fields, precentral ventral cortex], the Fronto-Parietal Control Network [parietal cortex (corresponding to the posterior division of the supramarginal gyrus), temporal cortex (corresponding to the posterior division of the middle temporal gyrus), dorsal prefrontal cortex, lateral prefrontal cortex, orbitofrontal cortex, ventral prefrontal cortex, medial posterior prefrontal cortex, precuneus, and the cingulate cortex], the Limbic Network [orbitofrontal cortex (corresponding to the frontal pole), temporal pole], the Somatomotor Network [somatomotor cortex (corresponding to the precentral gyrus)], and the Visual Network [visual cortex (corresponding to the superior division of the lateral occipital cortex)].

### Feature Weight Calculation

For feature weight generation and deployment, a final model was trained on features of all participants to derive overall classification weights. Specifically, the classification weights generated through feature selection were based upon the model parameters learned by the optimization function only during the training phase, *cf.* Equation (3). These weights can be applied to independent data from a new participant to predict their cognitive decline status associated with specific observed T1 and functional connectivity features in test data. The feature weights at each voxel, representing the relative contribution of each voxel to the classification, were separated by positive and negative weights that predict cognitive stability and decline, respectively (Cole et al., 2015). Positive and negative weights were divided by their respective sum of weights to compute the percent contribution of each voxel toward either positive or negative predictions. To demonstrate specific brain regions that predict decline to dementia, ROIs were defined using the 51 Yeo Atlas parcellations and ranked based on their average voxel percent contribution. Since features are selected based on the training data, the number of features varies per fold and data type.

## Results

A total of 55 participants met study criteria and were utilized for this secondary data analysis. Mean age of the participants was 72.5 (SD 7.7); the average educational attainment was 15.0 years (SD 3.14). The mean MoCA score was 22.0 (SD 3.0) (Table 1). Of the 232 potential participants who were excluded, 152 were excluded because they did not have aMCI at baseline (or had multiple diagnoses), 44 did not have valid T1 *and* rsfMRI baseline scans, 30 were missing the follow-up visit that came after the MRI visit, and 6 had aberrant QC metrics of rsfMRI data at baseline. Forty-one participants remained diagnostically stable over the study period and 14 declined to dementia at the most proximal follow-up evaluation to baseline. Of these 55 participants, 28 had fully complete neuropsychological battery test results, while 27 subjects had between one and two missing neuropsychological measures determined in *post-hoc* analyses to be missing at random (MAR). Participants who met criteria for multi-domain aMCI at baseline were significantly more likely to decline to dementia at follow-up when compared to participants with single-domain aMCI (chi-square = 5.85; odds ratio = 6.5; *p* = 0.016) (Table 1). Otherwise, groups did not differ significantly on any of the examined demographic or clinical factors.

**FIGURE 1:**
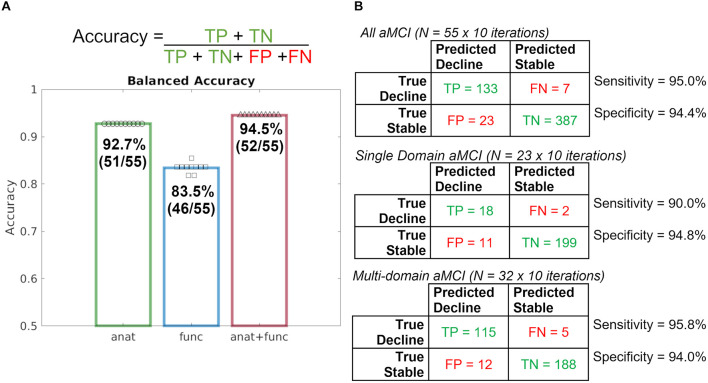
Repeated, nested, cross-validation test accuracy results for the prediction of aMCI patient (*N* = 55) diagnostic change at follow-up using baseline MRI alone, where a leave-one-out approach was used to predict whether or not a patient declined to dementia. **(A)** Accuracy formula and case predictions for each imaging modality overlaid with balanced case accuracy values. **(B)** Confusion matrices for all aMCI patients, Single Domain aMCI patients, and Multi-domain aMCI patients and their respective sensitivity and specificity values. *anat = T1; func = rsfMRI.*

Results of a repeated, nested cross-validation reveal a total accuracy for predicting diagnostic change at follow-up was 92.7% using baseline T1 imaging alone, 83.5% using rsfMRI alone, and 94.5% when combining T1 and rsfMRI modalities (Figure 1). As such, 51 of 55 participants were accurately classified using T1, 46 of 55 participants were accurately classified using rsfMRI, and 52 of 55 were accurately classified using both modalities together.

Given the significant statistical difference in the rate of single- and multi-domain aMCI across stable and decline groups, follow-up model performance investigation was completed. Follow-up investigation of single-domain and multi-domain aMCI subgroups revealed nearly identical model performance such that the combined T1 and rsfMRI achieved 94.4% total accuracy in the single-domain aMCI subgroup and 94.7% total accuracy in the multi-domain aMCI subgroup. These results indicate that although the prevalence of aMCI subtypes differs, the performance of the full, original model is comparable, and further model performance metrics will be calculated using all 55 participants together.

**FIGURE 2:**
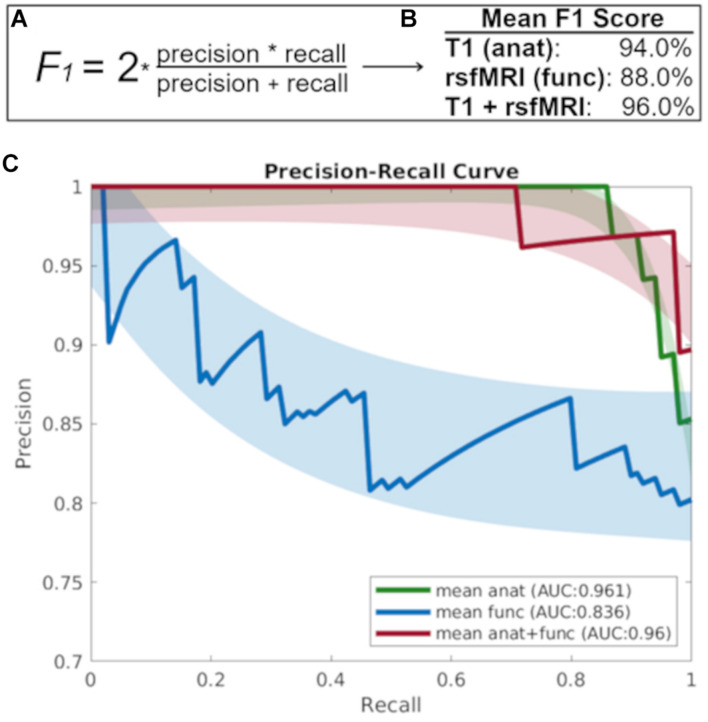
**(A)** The F1 score takes into account both precision and recall to measure model accuracy while accounting for false positives and false negatives. **(B)** The mean F1-score which gives more weight to false negatives and false positives while not allowing large numbers of true negatives influence the score, and **(C)** The precision recall curve focuses on the ability of each baseline imaging modality to predict diagnostic decline at follow-up. *anat = T1; func = rsfMRI.*

Figure 2 shows that the baseline T1 image alone is highly discriminant in the prediction of diagnostic decline as evidenced by a precision recall area under the curve (AUC) value 0.961, while the AUC for the baseline rsfMRI prediction was 0.836, and the combined T1 and rsfMRI model was 0.960. Further, the mean F1 score for each modality at the prediction of diagnostic decline was greater than 90% (94% for T1 alone, 88% for rsfMRI, and 96% for the multi-modal T1 and rsfMRI model). Lastly, results indicate an MCC value of 0.84 for the T1 image alone, an MCC value of 0.65 for the rsfMRI image alone, and an MCC value of 0.88 for the combination of T1 and rsfMRI.

### Supervised Machine Learning Feature Weight Classification

**FIGURE 3:**
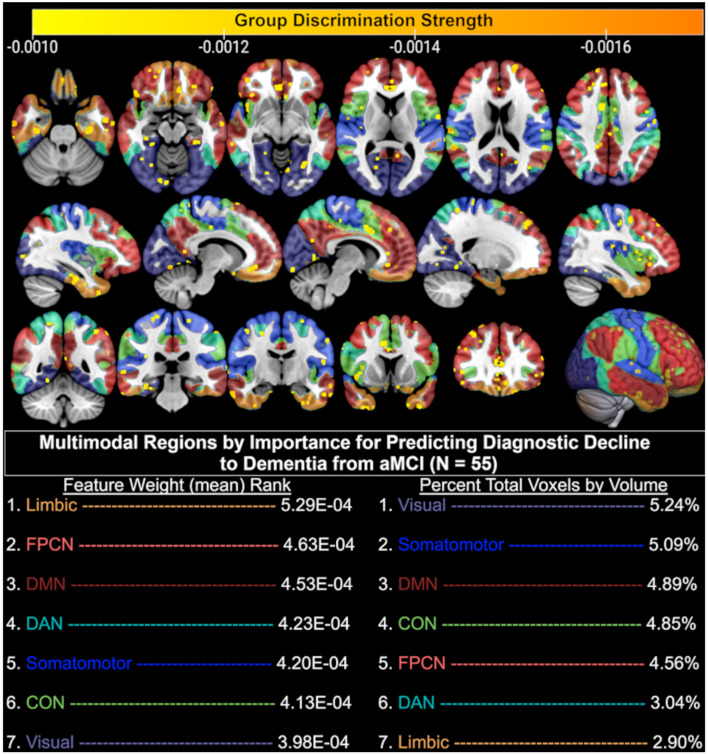
Brain regions (yellow-orange scale) where combined structural (T1) and functional (resting-state fMRI) MRI baseline data significantly discriminated between aMCI patients who remained diagnostically stable and those who declined to dementia at follow-up. Only the top 50% of significant (*p* < 0.01) voxels are displayed based on discrimination strength (voxels with weights 0.000 through –0.0009 excluded for visualization purposes); CON, Cingulo-opercular (Salience) Network; DAN, Dorsal Attention Network; DMN, Default Mode Network; FPCN, Fronto-parietal Control Network.

In the present dataset, T1-weighted data averaged 331,945 features, functional data averaged 85 features, and the combination of these data averaged 331,860 features. Given that a linear classifier was used to discriminate between aMCI patients who remained stable and declined diagnostically at the follow-up visit immediately after their baseline MRI, each feature in a given MRI sequence influenced this classification via its weight. As such, the larger the absolute magnitude of a given feature’s weight, the more strongly it influenced the optimal participant discrimination. As seen in Figure 3, this weight vector is projected onto the MNI-registered brain in order to display how strongly a given region of the brain influenced the optimal discrimination between aMCI patients with stable vs. declined diagnostic classification at follow-up. Given that combined structural and resting-state features were most predictive of diagnostic decline (see Figure 2), we developed a deployable model of combined structural and functional feature weights to demonstrate the importance of each brain region for the prediction of diagnostic decline. As such, we calculated a classifier for the group pairing (stable vs. decline) based on the MNI-registered normalized combination of the T1 voxelwise intensity values and the rsfMRI connectivity matrix using a *t*-test filter (*p* < 0.01) to select features with a significant difference between groups (Figure 3). Results indicate that when considering the mean feature weight of each multimodal region for predicting diagnostic decline, the limbic system results in the highest degree of group separation, yet the lowest percentage of total significant voxels by volume (2.90%). Conversely, the visual system demonstrated the lowest degree of group separation by feature weight, yet the highest total voxels by volume (5.24%). Rankings of remaining multimodal regions can be seen in Figure 3.

When examining baseline neuropsychological testing data alone, use of a class-mean filling approach to deal with missing data resulted in overall poor accuracy (60% repeated, nested, cross-validated test accuracy) for the prediction of decline from aMCI to dementia at follow-up (*N* = 55). To assess the effect of class-mean filling, observations with missing data were removed and the above analyses were repeated on the 28 subjects with complete data, yielding a 82.1% repeated, nested, cross-validated test accuracy for the prediction of decline from aMCI to dementia at follow-up. Lastly, when combining the class-mean filled neuropsychological data with the functional and anatomical model, nearly identical performance was observed to the multimodal imaging model, including a repeated, nested, cross-validated test accuracy of 94.5%, a mean AUC of 0.96, MCC of 0.88, and F1-Score of 0.96. However, when utilizing only those subjects with complete neuropsychological data (*N* = 28) along with multimodal neuroimaging data, repeated, nested, cross-validated test accuracy was 82.1%. In other words, in a limited subset sample of 28 participants, dementia conversion prediction based on neuropsychological performance is not improved by the addition of neuroimaging into the model. However, in a larger sample of 55 participants, multi-modal neuroimaging provides the greatest predictive ability and the model is not improved further by the addition of neuropsychological data.

## Discussion

The present study demonstrates that with limited baseline data (<45 min total MRI protocol), a multi-modal SVM model could predict diagnostic decline from amnestic MCI to dementia with over 94% accuracy and 96% precision. In fact, we show that 92.7% accuracy and 96% precision was achieved with a < 10 min T1 alone, and that neuroimaging outperformed a cognitive battery for predicting future decline. This finding has considerable clinical significance as it demonstrates the ability of easily obtained objective biomarkers to provide accurate and precise predictions about which high-risk aMCI patients will go on to develop dementia or potential Alzheimer’s disease (AD). While one other study has achieved a similar predictive accuracy in an MCI population also using MRI alone (Hojjati et al., 2018), the present study investigates higher risk aMCI patients and offers unique neuroanatomical information about the combined structural and functional regions that were optimally discriminative for the conversion to dementia in a relatively short time frame. Given the focus on amnestic MCI patients, the findings here provide a potentially stronger link to the increased incidence of AD as opposed to an all-MCI cohort, where true AD conversion rates are considerably lower (Ganguli et al., 2015, 2019; Oltra-Cucarella et al., 2018).

The performance of structural T1 MRI alone as a predictor for diagnostic decline to dementia was quite strong, and suggests that structural features were the largest driving force in both the prediction model as well as the resulting feature weights that separated stable patients from converters. This may in part be due to the use of all structural voxels in the model rather than restriction to known ROIs generated by outside parcellation algorithms, as has been done in prior MCI-to-dementia conversion prediction studies (Hojjati et al., 2018). Generation of multimodal feature weights, or combined neural regions that were optimally discriminative for the separation of aMCI patients who did and did not progress to dementia, revealed several important findings. We found that the combined structural and functional regions underlying the limbic system were the smallest in relative size after thresholding for significance, yet showed the strongest degree of group separation as seen by the mean feature weight of significant voxels in that region. Neuroanatomical regions within the limbic system, as defined by the Yeo atlas, include the orbitofrontal cortex and temporal pole. Similarly strong group separation performance was achieved by the regions underlying the frontoparietal control network (FPCN), which encompasses the anatomical regions of the temporal lobe, prefrontal cortex, cingulate cortex, and precuneus. These findings are highly consistent with the meta-analytic literature that suggest neural regions in the medial temporal lobe are associated with the progression from aMCI to AD (Ferreira et al., 2011), that alterations of the trans-entorhinal limbic regions are seen in MCI patients who eventually convert to AD (Schroeter et al., 2009), and that hypoactivation of the FPCN is observed in MCI patients relative to controls (Li et al., 2015).

We also found the combined structural and functional features underlying the default mode network to be highly important for dementia conversion prediction. This is consistent with the findings of a large meta-analysis in which MCI patients demonstrated hypoactivation in the default mode network, frontoparietal, and visual networks relative to healthy controls (Li et al., 2015). Feature weight generation also revealed that a large number of voxels in the multimodal regions underlying the visual system were significantly predictive of dementia conversion. This is consistent with meta-analytic work suggesting that relative to controls, both MCI and AD patients demonstrate hypoactivation of the visual system during rsfMRI (Li et al., 2015). We propose this finding may be related to degree of impairment, as more impaired patients are more likely have difficulty disengaging their visual systems during a resting paradigm and are thus more likely to fixate on the in-scanner screen. Regardless, this finding may be of clinical utility, as it in itself may be akin to a pathognomonic sign predictive of risk for dementia conversion. It is noted that the slightly higher accuracy of the combined structural-functional model for dementia conversion prediction may be a factor of the previously demonstrated reduced resting-state connectivity among MCI patients who convert to dementia compared to those who do not (Li Y. et al., 2016). A prior study used SVM to predict dementia conversion in an MCI-only cohort and found slightly lower predictive utility of sMRI than the present study (91% vs. 94% accuracy), higher predictive utility of rsfMRI (93% vs. 84% accuracy), and nearly identical combined predictive utility (97% vs. 96% accuracy). Differences observed in the predictive accuracy of rsfMRI may be the result of the use of different atlases for identification of functional connectivity data. Hojjati et al. (2018) utilized the 160-region Dosenbach rsfMRI atlas, while the present study utilized the Yeo 7-network atlas (Thomas Yeo et al., 2011). While not explicitly stated in their work, it is presumed that the interconnection of these 160 regions resulted in a total of 12,720 unique connections (using the formula: $x=\frac{n\,\left(n-1\right)}{2}$) available for predictive analyses, whereas the present study was limited to 1,275 unique connections. Thus, the inclusion of a higher number of data points appears to be, at least in part, a potential driver of higher accuracy in the SVM model. To this end, the present study’s higher accuracy for sMRI may also be driven by the use of all potential viable white and gray matter voxels in the brain rather than restriction to ROIs generated by an automated parcellation program (e.g., Freesurfer). As such, future work may seek to utilize a greater number of rsfMRI internodal connections to increase predictive ability of the uni- and multi-modal models.

With respect to the use of neuropsychological tests as predictors of future diagnostic decline, we found that the combination of six cognitive measures in an SVM model demonstrated the lowest accuracy of all predictor variables in the present study. This is particularly intriguing, as many of these cognitive measures were used in the formulation of the very consensus diagnosis being predicted, and we hypothesized it to have much higher predictive value for that reason. However, we note our own human nature lends to the ability to ignore missing data and maintain the goal of diagnosis, whereas a machine (SVM) does not deal well with such missing data. It is common to encounter missing neuropsychological test data in human studies, which certainly contributed to a smaller sample size and likely to the lower performance of the neuropsychological SVM model. While our model includes a disproportionately larger number of neuroimaging based features (331,945 structural features, 85 rsfMRI features) in comparison to the six neurocognitive measures in the combined model, if the neurocognitive measures were to have true predictive value, they would have been assigned a weight reflective of such. The fact that in the full 55-subject sample the model did not improve with their addition suggests neuroimaging likely outperforms these six neurocognitive measures for the prediction of future decline. Future work should aim to ensure all cases used for MRI prediction also retain full neuropsychological data to avoid the effects of missing data on SVM analyses.

The present single-center study has several limitations. First, only 14 participants progressed to dementia over a relative short time period, and slightly over half of these individuals were amyloid positive compared to a much lower amyloid positive rate in our cognitively normal individuals. While not statistically significant, those who converted to dementia tended to be older, have lower cognitive performance at baseline, and a higher rate of amyloid positivity; all of which suggest they may have been at an increased risk of conversion even apart from the MRI findings. Relatedly, we found a higher rate of conversion to dementia in a sub-group of participants with multi-domain amnestic MCI, though follow-up analysis determined the model equally successful at predicting dementia conversion within the two subgroups. Further, baseline neuroimaging outperformed a baseline battery of cognitive performance measures in the prediction of diagnostic decline at follow-up. Thus, we believe the high degree of accuracy and specificity in larger group prediction suggests our model is successful at identifying *individuals* who would ultimately decline to dementia despite the sample heterogeneity. With a larger sample, use of a single-domain aMCI only population may have led to stronger feature weights in the regions ultimately implicated in Alzheimer’s disease, such as the limbic system and temporal lobe regions. We used cross-validation strictly for model performance reporting and not for building a final deployment model. As such, the final model was trained on all subjects and was only used to generate weight maps for deployment to make predictions about new, future data. Future studies are required to determine whether our final algorithm could as accurately predict additional cases from our cohort and importantly to different cohorts such as the Alzheimer’s Disease Neuroimaging Initiative (ADNI) and other datasets to assess generalizability of results. While we found that the performance of the combined structural and functional model exceeded the performance of each modality alone, it did so by only a small percentage when compared to the unimodal T1 model. While our data do not provide the ability to comment on how the strength of rsfMRI activation in the important networks influenced multimodal predictive accuracy, our future work aims to investigate the patterns of hyper- and hypo-activation in these identified networks and compare between patients who do and do not convert to dementia. Lastly, in the future, we seek to determine the utility of this model to predict conversion to MCI in a population of healthy older adults with no evidence of MCI but with subjective cognitive complaints.

## Conclusion

We demonstrate that a combination of structural and functional information undetected by the human eye can be used to accurately identify high-risk amnestic MCI patients who will develop dementia a short time later. The model deployed in this study independently revealed that several neuroanatomical regions commonly implicated in the development of Alzheimer’s disease were the largest drivers in identifying amnestic MCI patients who progress to dementia at follow-up. Further evaluation of our model with larger cohorts, longer follow-up periods, evaluation of amyloid load, and diverse ethnic and cultural groups have the potential to advance the field. If further validated, this technique has the potential to contribute to the identification of individuals with aMCI who are at high risk of progression to dementia, and thus could be prioritized for studies targeting disease modification.

## Data Availability Statement

The datasets presented in this article are not readily available because of restrictions imposed by the administering and funding institutions given the ongoing nature of the funded project, the data utilized for this manuscript are unavailable for public hosting. Upon completion of this funding period, data can be made available by request to the authors given that a formal data sharing agreement is signed by the requesting agency. All software and code used in the present manuscript is freely available to the public. Requests to access the datasets should be directed to RD, duara@msmc.com.

## Ethics Statement

Participants were recruited through the 1Florida Alzheimer’s Disease Research Center (ADRC) for an IRB-approved longitudinal investigation performed in accordance with the declaration of Helsinki (P50-AG047266-05). The patients/participants provided their written informed consent to participate in this study.

## Author Contributions

JG: conceptualization, methodology, formal analysis, visualization, investigation, writing-original draft, writing-editing, and funding acquisition. AA: methodology, formal Analysis, investigation, visualization, software, and writing-original draft. RF: methodology and formal analysis. DL and RD: project administration, supervision, and writing—review and editing. MR, MA, TR, and SD: project administration and writing—review and editing. HH: formal analysis, writing-original draft, and writing—review and editing. AW: formal analysis, conceptualization, writing—review and editing, and supervision. RC: writing—review and editing, and supervision. All authors contributed to the article and approved the submitted version.

## Conflict of Interest

The authors declare that the research was conducted in the absence of any commercial or financial relationships that could be construed as a potential conflict of interest.

## Publisher’s Note

All claims expressed in this article are solely those of the authors and do not necessarily represent those of their affiliated organizations, or those of the publisher, the editors and the reviewers. Any product that may be evaluated in this article, or claim that may be made by its manufacturer, is not guaranteed or endorsed by the publisher.
